# Impact of frequency of application on the long-term efficacy of the biocontrol product Aflasafe in reducing aflatoxin contamination in maize

**DOI:** 10.3389/fmicb.2022.1049013

**Published:** 2022-11-24

**Authors:** Joseph Atehnkeng, Peter S. Ojiambo, Alejandro Ortega-Beltran, Joao Augusto, Peter J. Cotty, Ranajit Bandyopadhyay

**Affiliations:** ^1^Pathology and Mycotoxin, International Institute of Tropical Agriculture (IITA), Ibadan, Nigeria; ^2^Center for Integrated Fungal Research, Department of Entomology and Plant Pathology, North Carolina State University, Raleigh, NC, United States; ^3^College of Food Science and Engineering, Ocean University of China, Qingdao, China; ^4^Agricultural Research Service, United States Department of Agriculture, Tucson, AZ, United States

**Keywords:** maize, aflatoxins, atoxigenic isolates, biocontrol, carry-over

## Abstract

Aflatoxins, produced by several *Aspergillus* section *Flavi* species in various crops, are a significant public health risk and a barrier to trade and development. In sub-Saharan Africa, maize and groundnut are particularly vulnerable to aflatoxin contamination. Aflasafe, a registered aflatoxin biocontrol product, utilizes atoxigenic *A. flavus* genotypes native to Nigeria to displace aflatoxin producers and mitigate aflatoxin contamination. Aflasafe was evaluated in farmers’ fields for 3 years, under various regimens, to quantify carry-over of the biocontrol active ingredient genotypes. Nine maize fields were each treated either continuously for 3 years, the first two successive years, in year 1 and year 3, or once during the first year. For each treated field, a nearby untreated field was monitored. Aflatoxins were quantified in grain at harvest and after simulated poor storage. Biocontrol efficacy and frequencies of the active ingredient genotypes decreased in the absence of annual treatment. Maize treated consecutively for 2 or 3 years had significantly (*p* < 0.05) less aflatoxin (92% less) in grain at harvest than untreated maize. Maize grain from treated fields subjected to simulated poor storage had significantly less (*p* < 0.05) aflatoxin than grain from untreated fields, regardless of application regimen. Active ingredients occurred at higher frequencies in soil and grain from treated fields than from untreated fields. The incidence of active ingredients recovered in soil was significantly correlated (*r* = 0.898; *p* < 0.001) with the incidence of active ingredients in grain, which in turn was also significantly correlated (*r* = −0.621, *p* = 0.02) with aflatoxin concentration. Although there were carry-over effects, caution should be taken when drawing recommendations about discontinuing biocontrol use. Cost–benefit analyses of single season and carry-over influences are needed to optimize use by communities of smallholder farmers in sub-Saharan Africa.

## Introduction

Aflatoxins are highly toxic metabolites produced by some members of *Aspergillus* section *Flavi* before, during, and after harvest (Cotty et al., 1994) when plants are infected in the field. High temperature, soil moisture stress, and insect injury influence the quantities of aflatoxins accumulated in susceptible crops (Amaike and Keller, 2011). Aflatoxins can cause cancer, liver disease, and death. These toxins are also associated with growth faltering and immune system suppression (JECFA, 2018; Ismail et al., 2021). In sub-Saharan Africa (SSA), including Nigeria, human exposure to aflatoxin is high (JECFA, 2018). Aflatoxins commonly contaminate several staple cereals, root crops, and groundnut, which form the agricultural, economic and food security backbone of the region (Gnonlonfin et al., 2013; Udomkun et al., 2017a). Productivity of livestock is also impacted through consumption of contaminated feed (da Rocha et al., 2014; Monson et al., 2015). Many nations impose regulations to prevent consumption of contaminated commodities and by doing so, may reduce human and animal exposure to aflatoxins (Wu, 2015; Sirma et al., 2018). However, in the developing world, enforcement of such regulations is inadequate to prevent ingestion of dangerous concentrations of aflatoxins (Matumba et al., 2017).

Aflatoxin-producing fungi are diverse. The most common aflatoxin producer, *A. flavus*, can be subdivided into two broad groups with either S- or L-morphology (or morphotype), both producing only the B aflatoxins (Cotty, 1989; Singh et al., 2020). S-morphotype fungi produce numerous small sclerotia (avg. diameter < 400 μm) and high aflatoxin levels. On the other hand, L-morphotype fungi produce fewer, larger sclerotia (diameter >=400 μm) and, on average, lower aflatoxin concentrations. The most prevalent aflatoxin-producing species in Nigeria is *A. flavus* (Bandyopadhyay et al., 2016), with isolates primarily belonging to the L-morphotype. Within the L-morphotype of *A. flavus*, there are isolates that do not produce aflatoxins (i.e., atoxigenic; Mehl et al., 2012). Each morphotype is further subdivided into many vegetative compatibility groups (VCGs) delineated by a heterokaryon incompatibility system (Leslie, 1993; Atehnkeng et al., 2016). Some VCGs contain only atoxigenic members and these atoxigenic VCGs can be used as biocontrol agents to outcompete aflatoxin-producers and reduce aflatoxin in crops (Mehl et al., 2012). Atoxigenic isolates used as biocontrol agents cannot produce aflatoxins due to defects in the aflatoxins biosynthesis gene cluster (Chang et al., 2005; Donner et al., 2010; Adhikari et al., 2016).

The *A. flavus* S-morphotype is an important aflatoxin producer in several regions but has not been reported in West Africa (Probst et al., 2014; Frisvad et al., 2019). On the other hand, a group of the S-morphotype fungi, initially identified as unnamed taxon S_BG_, produce both B and G aflatoxins and are associated with several crops in West Africa (Cotty and Cardwell, 1999; Atehnkeng et al., 2008a; Diedhiou et al., 2011; Agbetiameh et al., 2018; Ezekiel et al., 2019). This unnamed taxon S_BG_ is an important causal agent of contamination even if occurring at low frequencies. Recently, isolates of this unnamed taxon S_BG_ isolates were delineated into *A. aflatoxiformans*, *A. austwickii*, *A. cerealis*, *A. minisclerotigenes*, and unknown taxa based on both DNA and extrolite profiles (Probst et al., 2014; Frisvad et al., 2019; Singh and Cotty, 2019; Singh et al., 2020). In the current paper, as in other papers not utilizing DNA-based phylogenetics (Ezekiel et al., 2019), we use the term S_BG_ species for all S-morphotype fungi that produce both B and G aflatoxins.

Production of aflatoxins typically begins in the field during crop development and continues during harvest and post-harvest stages (Cotty et al., 1994; Waliyar et al., 2014; Seetha et al., 2017; Mahuku et al., 2019). Therefore, management programs to prevent aflatoxin contamination must be effective in the field, and include technologies that provide protection until crop utilization (Mehl et al., 2012). Several cultural, biological, mechanical, and processing strategies can aid in the management of aflatoxins at pre- and post-harvest stages (Matumba et al., 2015; Ayalew et al., 2017; Udomkun et al., 2017b; Ojiambo et al., 2018; Walker et al., 2018). Stress-tolerant varieties, good agronomic practices, use of biocontrol products, adequate grain drying, proper hermetic storage, sorting, among others, are methods that can reduce aflatoxin contamination and these options are more effectice when used in combination.

Aflatoxin biocontrol products have been used in the US for over 20 years because of large reductions in aflatoxins following treatment (Cotty, 2006; Mehl et al., 2012; Doster et al., 2014; Weaver et al., 2015; Ortega-Beltran and Bandyopadhyay, 2019). Two aflatoxin biocontrol products are registered with the US Environmental Protection Agency (USEPA), one for use in cotton, maize, pistachio, almond, and fig, and another for use in maize and groundnut (USEPA, 2003, 2016, 2017). The International Institute of Tropical Agriculture (IITA) in collaboration with the US Department of Agriculture – Agricultural Research Service (USDA–ARS; the developer of the original technology) and partners, optimized the aflatoxin biocontrol technology for use in SSA (Bandyopadhyay et al., 2016). Several biocontrol products under the tradename Aflasafe have been developed for use in various SSA countries. Each Aflasafe product contains four active ingredient atoxigenic *A. flavus* isolates each belonging to distinct atoxigenic African *A. flavus* VCGs (AAVs) native to, and widely distributed in the target nation (Moral et al., 2020; Bandyopadhyay et al., 2022). The first aflatoxin biocontrol product in Africa was developed for use in Nigeria and registered in 2014 with the National Agency for Food and Drug Administration and Control (NAFDAC) of Nigeria for use on maize and groundnut. A 10-year study showed that the original Aflasafe product is highly effective at limiting aflatoxin contamination in farmers’ fields at harvest and during post-harvest period when used on a commercial scale (Bandyopadhyay et al., 2019).

Country specific Aflasafe products have been very effective at limiting aflatoxin contamination of chili peppers (Ezekiel et al., 2019), maize and groundnut (Bandyopadhyay et al., 2019; Narayan et al., 2019; Schreurs et al., 2019). This includes experience in the countries Ghana, Senegal, The Gambia, Burkina Faso, and Nigeria in West Africa (Bandyopadhyay et al., 2019; Agbetiameh et al., 2020 Senghor et al., 2020, 2021), Tanzania and Kenya in East Africa (Bandyopadhyay et al., 2016; Mahuku et al., 2022) and Malawi, Zambia, and Mozambique in Southern Africa (Moral et al., 2020; Bandyopadhyay et al., 2022). However, several aspects of the technology remain unknown, including how often fields have to be treated. The current recommendation is to treat crops with an aflatoxin biocontrol product once every cropping cycle.

Farmers and authorities in Nigeria and elsewhere question if it is necessary to apply aflatoxin biocontrol products on a yearly basis. The same question has been raised by several researchers (Ehrlich et al., 2015; Gressel and Polturak, 2018; Njoroge, 2018; Kagot et al., 2019; Molo et al., 2019; Pitt, 2019; Andrews et al., 2020). Continuous treatment may result in unnecesasry expenses for farmers who use biocontrol products in Africa and elsewhere. Although carry-over between cropping seasons has been quantified in large-scale mechanized agriculture (Cotty, 2000, 2006; Jaime et al., 2017), carry-over of active ingredients between cropping seasons in smallholder fields in Africa has not been carefully quantified. Therefore, the main objective of the current study was to determine how frequently an aflatoxin biocontrol product should be applied to farmers’ fields in Nigeria by assessing (i) carry-over of biocontrol active ingredients after single season or multiple seasons applications, and (ii) influences of biocontrol product applications on aflatoxin content of maize during the year of application and during subsequent years. Results from the current three-year study provide information that may be valuable for the development of cost-effective aflatoxin management strategies utilizing atoxigenic strain-based biocontrol products in smallholder agriculture across Nigeria and elsewhere.

## Materials and methods

### Biocontrol product and its formulation

The biocontrol product Aflasafe contains as active ingredients four atoxigenic genotypes of the *A. flavus* L-morphotype native to Nigeria: La3279, La3304, Ka16127, and Og0222. Each isolate belongs to an AAV containing only atoxigenic genotypes. The active ingredients were originally isolated from maize grain in Nigeria between 2003 and 2005 (Atehnkeng et al., 2016). Aflasafe is currently manufactured commercially using an industrial process (Bandyopadhyay et al., 2016, 2022). However, for the current study, the product was prepared with a previously described laboratory-scale process (Atehnkeng et al., 2014).

### Study locations and biocontrol treatment combinations

Impacts of biocontrol applications were investigated in maize fields of smallholder farmers who agreed to participate in the experiments. Farmers followed agronomic practices typical of their area. Experiments were conducted in Lere, Maigana, and Birnin Gwari in the state of Kaduna, Nigeria, during the rainy season when temperature during the maize growing period ranged from 22 to 31°C. These three localities fall within a 200-km^2^ radius of each other. Treatments were: (i) a single application in 2009, (ii) applications in 2009 and 2010, (iii) applications in 2009 and 2011, and (iv) applications in 2009, 2010, and 2011. For each treatment, a total of nine maize fields and nine paired untreated fields were used. The maize fields were typical smallholder farmers’ fields in Kaduna State. Field size ranged from 0.25 to 2 ha. The Aflasafe product was broadcast manually by hand at 10 kg/ha, 2-to-3 weeks before flowering. Untreated fields were separated by at least a 500-m buffer from treated fields.

### Sampling and processing of soil and maize

At harvest, maize plants were cut at the base, as is typical in this region, and stalks with ears were stacked upright in the field in the form of a cone containing approximately 30 plants as per farmers’ practice. Twenty-five maize ears were randomly collected from cones 2–3 weeks after harvest. The collected ears were transported to the IITA Pathology and Mycotoxin Laboratory and subdivided into two sets: one set with 20 ears and a second with 5 ears. Ears in the first set were de-husked, shelled, and grains were stored at 4°C before processing for analyses. For each sample in the first set, 250 g of grain was ground using a blender (Waring Commercial, Stamford, CT) to <20 μm-sized particles. The blender cup was washed with 80% ethanol between samples to prevent any cross contamination.

The set of 5 ears was subjected to conditions that accelerate maize spoilage. Briefly, the ears were soaked in water for 3 h in polythene bags. After draining, the ears were left to dry on a flat, dry, clean surface inside a screenhouse. Maxima and minima temperatures ranged from 35 to 42°C and 22 to 28°C, respectively. After 7 days, maize ears were de-husked, shelled, and processed for analyses as described above.

Each year, soil samples were also collected from all fields at harvest to determine the proportion of *A. flavus* belonging to the applied AAVs. Soil (about 150 g) was collected along a transect by taking 40 to 50 subsamples from three random locations to a depth of 2-cm (Cotty, 1997). Upon arrival in the laboratory, samples were dried in a forced air oven (2 days, 50°C) and transferred to into polythene bags in a biosafety cabinet where clods were eliminated with a hammer and samples were homogenized.

### Aflatoxin analyses

Aflatoxins were extracted by blending a 20-g ground maize sample with 50 ml 70% methanol for 3 min (Atehnkeng et al., 2008b). The mixture was filtered with Whatman No. 1 filter paper (Whatman Intl. Ltd., Maidstone, England) and the filtrate collected in a 250-ml separation funnel. The solution was combined with 25 ml methylene chloride and thoroughly mixed. The methylene chloride partition was filtered through 40 g anhydrous sodium sulfate to remove residual water. The extraction was performed twice, and extracts were pooled and evaporated to dryness in a fume hood in the dark. The residue was dissolved in 1 ml methylene chloride and either diluted or concentrated to allow for accurate densitometry following separation of aflatoxins alongside aflatoxin standards (B_1_, B_2_, G_1_, and G_2_; Supelco, Bellefonte, PA) on thin-layer chromatography (TLC) plates (Silica gel 60, EMD, Darmstadt, Germany). Aflatoxins were quantified using a fluorescence scanning densitometer, CAMAG TLC Scanner 3 with WinCATS 1.4.2 software (Camag AG, Muttenz, Switzerland). Concentrations of aflatoxin B_1_ and aflatoxin B_2_ were summed and presented in this paper. Aflatoxin G_1_ and aflatoxin G_2_ were generally below the limit of detection and were not included in the calculations. The limit of detection was 1 part per billion (ppb). All chemicals used in this study were of Analytical Grade with more than 99% purity.

### Mycoflora analysis in soil and maize grain

Members of *Aspergillus* section *Flavi* were isolated from soil and grain with dilution plate technique on modified rose Bengal agar (MRBA; Atehnkeng et al., 2008a). Briefly, 1 g of sample was suspended in 10 ml sterile distilled water in a 40 ml glass vial, vortexed for 2 min, and appropriate dilutions were plated on MRBA. Plates were incubated in the dark (31°C, 3 days). Colony-forming units (CFU) per g were determined. Colonies from plates containing less than 10 *Aspergillus* section *Flavi* colonies were transferred to 5–2 agar [(5% V-8 juice (Campbell Soup Company, Camden, NJ), 2% Bacto-agar (Difco Laboratories Inc., Detroit, MI), pH 5.2] and incubated (31°C, 5 days). Isolates were classified as *A. flavus* L-morphotype, an S-morphotype species (grouped as S_BG_ species; Singh et al., 2020), *A. parasiticus*, or *A. tamarii* based on colony morphology, and sclerotial and conidial characteristics (Cotty, 1989; Agbetiameh et al., 2018).

### Vegetative compatibility group analysis

Frequencies of biocontrol AAVs in soil and grain at harvest were determined using VCG analyses, as previously described (Grubisha and Cotty, 2010; Atehnkeng et al., 2016). From each sample, 20 L-morphotype isolates were examined to assess membership in one of the four biocontrol AAVs using nitrate non-utilizing (*nit*^−^) mutants. Fungal suspensions (15 μl containing approx. 1,000 spores) of each biocontrol AAV tester mutant and the *nit*^−^ mutant of interest were seeded into individual 5 mm diameter wells 1 cm apart (in a triangular pattern) cut into starch agar [36 g/l dextrose, 20 g/l soluble starch, 2% Bacto-agar, pH 6.0; (Ortega-Beltran and Cotty, 2018)] and incubated (31°C, 7 days). Mutants of isolates complementing one or both of the biocontrol AAV testers to form prototrophic regions at the zone of mycelial interaction were assigned to that AAV.

### Data analysis

Fungal densities (CFU/g), frequency of *Aspergillus* spp. in soil, frequency of biocontrol AAVs in soil and grain, and aflatoxin content (ppb) in grain at harvest and after simulated poor storage (response variables, *x*) were transformed using the equation *y* = log_10_ (1 + *x*) to normalize variances. Data were subjected to statistical analyses using the mixed procedure (PROC MIXED) of SAS software v9.2 (SAS Institute, Cary, NC). The experiments were conducted in a randomized complete block design and each field was considered a replicate. Replicates were considered random effects, while treatments were considered fixed effects. Treatment means were separated for significance using paired Student *t*-tests (*α* = 0.05) as implemented in the SAS PROC TTEST.

## Results

### Aflatoxin concentration in grain from biocontrol-treated and untreated maize fields

At harvest, total aflatoxin content ranged from 1.1 ppb (3-year sequential application) to 6.0 ppb (single application in 2009) in maize grain from biocontrol-treated maize fields (Table 1). Total aflatoxin in grain from untreated maize fields ranged from 10.4 to 52.2 ppb. Aflatoxin reductions at harvest ranged from 52.6 to 95.6%. Grain from treated fields had significantly (*p* < 0.05) less aflatoxin than that from untreated fields except for fields treated only once in 2009. These fields had a significant reduction only in 2009, the year of treatment, but not in 2010 and 2011 (Table 1). However, in those two cases, the aflatoxin content in grain from untreated fields was relatively low (12.4 and 10.4 ppb, respectively).

**Table 1:** Aflatoxin concentration in maize kernels from farmers’ fields treated with different biocontrol application regimens in Kaduna State, Nigeria.

Year of treatment	Year of observation			Aflatoxin concentration (ppb)					
				At harvest			After simulated poor storage		
		Treatment^a^	*n*	Mean	Std. Error	Reduction (%)^b^	Mean	Std Error	Reduction (%)^2^
2009	2009	Treated	9	3.7*	1.67	83.7	20.9**	10.80	94.7
		Untreated	9	22.5	5.13		395.1	181.20	
2009	2010	Treated	9	5.0	1.43	59.8	12.4**	4.17	90.7
		Untreated	9	12.4	7.23		133.1	49.87	
2009	2011	Treated	9	6.0	2.23	52.6	90.0*	25.57	96.6
		Untreated	9	10.4	2.93		2,636.0	1587.33	
2009, 2010	2010	Treated	9	4.4**	0.93	91.6	25.9**	9.93	72.5
		Untreated	9	52.2	10.80		94.4	17.53	
2009, 2010	2011	Treated	9	2.1*	0.07	95.6	19.3**	0.37	94.5
		Untreated	9	48.1	0.27		353.0	1.47	
2009, 2011	2011	Treated	9	3.4*	1.53	82.9	86.7*	21.13	92.2
		Untreated	9	19.6	10.97		1,107.0	583.00	
2009, 2010, 2011	2011	Treated	9	1.1*	0.60	91.2	4.9**	1.40	98.1
		Untreated	9	12.2	3.03		256.0	53.90	

^a^Untreated: fields where Aflasafe was never applied; Treated: fields where Aflasafe was applied (10 kg/ha, 2–3 weeks before flowering) during the indicated year. ^b^Reduction (%) = ([aflatoxin mean of untreated maize – mean of Aflasafe-treated-maize]/aflatoxin mean of untreated maize) × 100. Significant differences between aflatoxin content in grain from treated and untreated fields are indicated by *p < 0.05 or ** < 0.01 based on Student’s *t*-test.

After subjecting maize cobs to poor storage, in all cases, grain from biocontrol-treated fields had significantly (*p* < 0.05) less aflatoxin than grain from untreated fields (Table 1). Total aflatoxin ranged from 4.9 ppb (3-year sequential application) to 90 ppb (single application in 2009) in grain from biocontrol-treated fields, and from 94 to 2,636 ppb in untreated maize. Biocontrol treatments resulted in aflatoxin reductions ranging from 72.5 to 98.1% (Table 1).

### *Aspergillus* section *Flavi* densities in soil and maize grain

Densities of *Aspergillus* section *Flavi* in soil ranged from 221 to 783 CFU/g in untreated fields, and from 110 to 850 CFU/g in biocontrol-treated fields (Table 2). Fungal density in soils treated only the first year was on average about 850 CFU/g but decreased when left untreated the following year (255 CFU/g) and further decreased when left untreated for 2-years (110 CFU/g). Fungal densities averaged >500 CFU/g after treatment in two successive years. However, these levels reduced to 150 CFU/g when left untreated the third year. Biocontrol treatment for 3 years did not increase *Aspergillus* densities in soil (254 CFU/g) compared to either a single treatment (850 CFU/g) or a 2 year sequential treatment (507 CFU/g; Table 2).

**Table 2:** Population densities of *Aspergillus* section *Flavi* in soil and grain from maize fields treated with diverse biocontrol regimens in Kaduna State, Nigeria.

Year of treatment	Year of observation	Treatment^a^		CFU/g^b^	
			*n*	Soil	Grain
2009	2009	Treated	9	850	2,785
		Untreated	9	783	2,074
2009	2010	Treated	9	255	8,294
		Untreated	9	328	8,369
2009	2011	Treated	9	110	177
		Untreated	9	221	76
2009, 2010	2010	Treated	9	507	4,003
		Untreated	9	270	4,642
2009, 2010	2011	Treated	9	150	200
		Untreated	9	331	91
2009, 2011	2011	Treated	9	394	51
		Untreated	9	675	40
2009, 2010, 2011	2011	Treated	9	254	33
		Untreated	9	708	79

^a^Untreated: fields where Aflasafe was never applied; Treated: fields where Aflasafe was applied (10 kg/ha, 2-to-3 weeks before flowering) during the indicated year. ^b^CFU/g: colony forming units per g of sample. No differences were detected in CFU/g in soil and grain from treated and untreated maize fields based on Student’s *t*-test (*p* = 0.05). CFU/g values were log transformed (log_10_[CFU/g + 1]) prior to analysis to stabilize the variance.

Fungal densities in grain ranged from 40 to 8,369 CFU/g in untreated maize, and from 33 to 8,294 CFU/g in biocontrol-treated maize (Table 2). In treated fields, fungal densities in grain decreased from 2,785 CFU/g after a single year treatment to 178 CFU/g when left untreated for two subsequent years. Similarly, fields with over 4,000 CFU/g after 2 years of treatment decreased to 200 CFU/g when left untreated the following year. Treating fields consecutively for 3 years did not increase grain fungal densities (33 CFU/g) compared to grain from fields treated only the first year (2,785 CFU/g) or sequentially for 2 years (4,003 CFU/g). Overall, fungal densities in either soil or grain from treated fields did not differ significantly (*p* > 0.05) from those of untreated fields (Table 2).

### Distribution of *Aspergillus* species in soil and maize grain

The *A. flavus* L-morphotype was the most abundant member of *Aspergillus* section *Flavi* in both soil and grain (Table 3). In soil, there was a slight decline that was not statistically significant (*p* = 0.05) in frequencies of the L-morphotype when fields were treated once (93.9%), and left untreated for 1 year (91.5%), and 2 years (89.7%). Conversely, the frequency of the highly toxigenic S_BG_ species in soil increased in the absence of treatment (Table 3). The S_BG_ species were not found in soil treated consecutively for 2 years, but S_BG_ species’ frequency increased (to 4.5%) in these fields after 1 year without treatment. Treatment of fields a second time after 1 year without treatment resulted in an average S_BG_ species’ frequencies of 2.4% which was not significantly different from any of the other treatments. *A. parasiticus* was not found in in either soil or grain during the 3-year study and *A. tamarii* was detected in soil from some untreated fields (up to 18.4%) but was sporadically detected in grain from either treated or untreated maize fields (range = 0 to 4.6%; Table 3). The L-morphotype was the sole fungus always detected in soil and grain from all fields (Table 3).

**Table 3:** Frequency of members of *Aspergillus* section *Flavi* in soil and grain from maize fields treated with diverse biocontrol regimens in Kaduna State, Nigeria.

Year of treatment	Year of observation			Frequency of *Aspergillus* species/strain (%)^b^						
		Treatment^a^		Soil				Grain		
			*n*	*A. flavus* L- morphotype	S_BG_	*A. tamarii*		*A. flavus* L- morphotype	S_BG_	*A. tamarii*
2009	2009	Treated	9	93.9	0.6	5.5		100	0	0
		Untreated	9	92.5	3	4.5		94.5	5.5	0
2009	2010	Treated	9	91.5	2.5	6		91.4	8.6	0
		Untreat	9	74.6	7	18.4		94.5	3.7	1.8
		ed								
2009	2011	Treated	9	89.7	5.7	4.6		98.7	1.3	0
		Untreated	9	92.2	6.4	1.4		92.2	6.8	1
2009, 2010	2010	Treated	9	95.9**	0.0**	4.1		100	0	0
		Untreated	9	79.2	15.2	5.6		92	8	0
2009, 2010	2011	Treated	9	95.5	4.5	0		100	0	0
		Untreated	9	93	7	0		100	0	0
2009, 2011	2011	Treated	9	97.6	2.4	0		100	0	0
		Untreated	9	92.7	5.8	1.5		90.8	4.6	4.6
2009, 2010, 2011	2011	Treated	9	100	0	0		100	0	0
		Untreated	9	97.5	0	2.5		100	0	0

^a^Untreated: fields where Aflasafe was never applied; Treated: fields where Aflasafe was applied (10 kg/ha, 2-weeks before flowering) during the indicated year. ^b^Frequency values, expressed as a percentage of the total section *Flavi* community, were log transformed (log_10_[*x* + 1]) prior to analysis to stabilize the variance. Significant differences between frequencies of each type fungi in soil and grain from treated and untreated maize fields are indicated by *P < 0.05 or **P < 0.01 based on Student’s *t*-test.

### Recoveries of biocontrol genotypes in soil and maize grain

**Figure 1:**
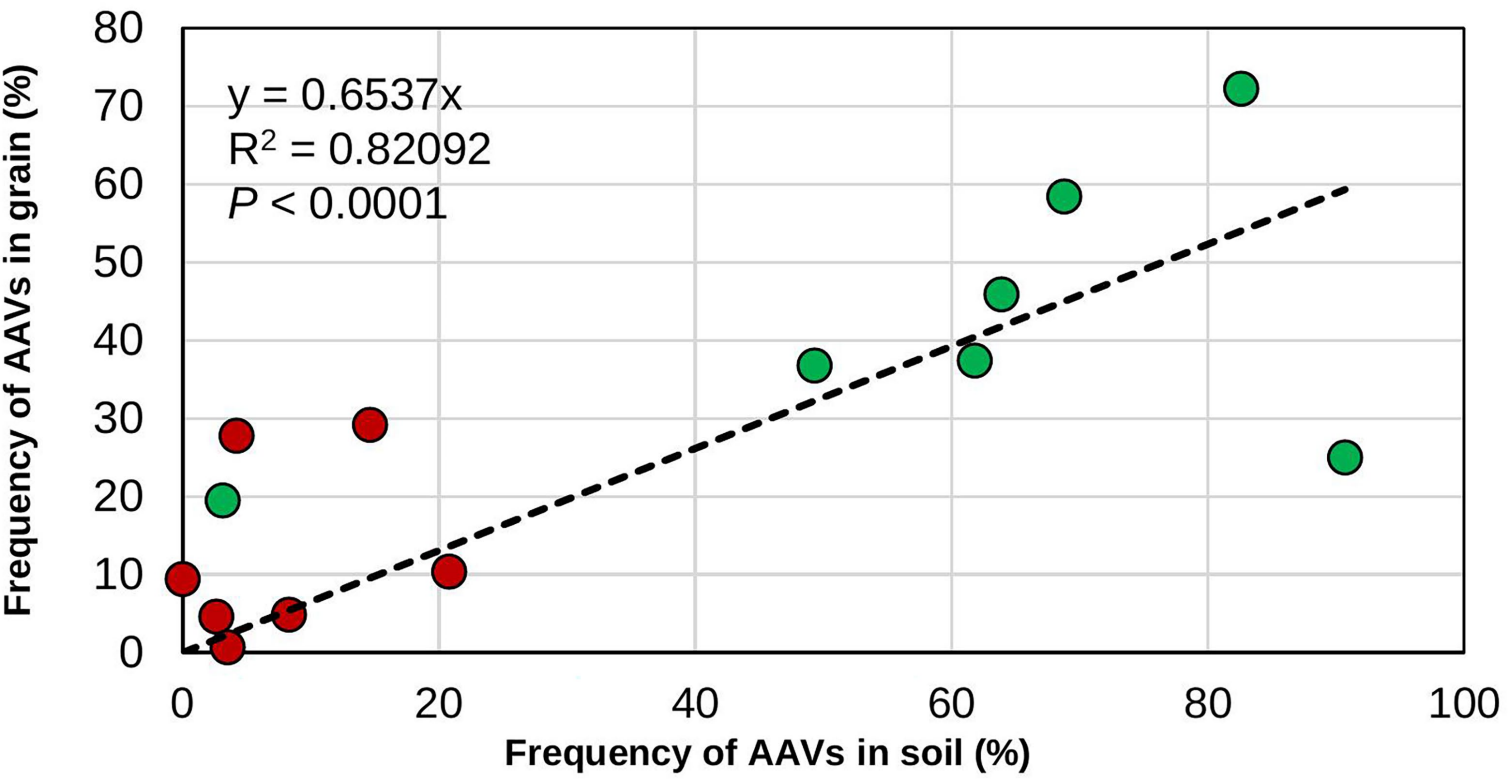
Relationship between frequency of the atoxigenic *Aspergillus flavus* vegetative compatibility groups (AAVs) composing the biocontrol product in soils and grains. The data are for the frequencies in soil and grain from both treated (green circles) and untreated (red circles) maize fields.

Generally, the recovery of biocontrol AAVs from treated fields was higher in soil than in grain (Table 4). In addition, biocontrol AAVs occurred at a higher (*p* < 0.05) frequency in soil and grain from treated fields than from untreated ones (Table 4). There was a gradual decrease in biocontrol AAVs’ frequency in soils from fields treated the first year (74.3%) and left untreated in the second (63.8%) and third year (40.3%). Similarly, in grain, biocontrol AAVs’ frequency decreased when fields were treated the first year (77.8%) and left untreated in the second (43.1%) and third year (36.1%). The 2-year sequential and alternate treatments yielded biocontrol AAVs frequency increasing from 65.0 to 87.5% in soil and from 62.5 to 70.6% in grain. A 3-year treatment resulted in biocontrol AAVs frequency in soil (72.9%) and grain (68.8%) that were within the range of the 2-year treatments, either sequential or alternate treatments (Table 4). In most cases, AAVs were detected in both grain and soil from untreated fields, although these levels (up to 27.8%) were markedly lower than those detected in treated fields. Overall, for both treated and untreated fields, the incidence of biocontrol AAVs recovered in grain was significantly correlated (*r* = 0.898; *p* < 0.001) with the incidence of biocontrol AAVs in soil (Figure 1). The proportion of biocontrol AAVs in grain was also significantly correlated (*r* = −0.621, *p* = 0.02) with aflatoxin concentration.

**Table 4:** Overall frequencies of Aflasafe genotypes in soil and grain from maize fields treated with diverse Aflasafe regimens in Kaduna State, Nigeria.

Year of treatment	Year of observation	Treatment^a^		Frequency of Aflasafe genotypes (%)^b^	
			*n*	Soil	Grain
2009	2009	Treated	9	71.6*	77.8**
		Untreated	9	13.2	17.4
2009	2010	Treated	9	63.8**	43.0**
		Untreated	9	7.5	4.4
2009	2011	Treated	9	40.2**	36.1
		Untreated	9	14.6	27.8
2009, 2010	2010	Treated	9	79.4 **	70.6**
		Untreated	9	4.4	0.6
2009, 2010	2011	Treated	9	87.5**	62.5**
		Untreated	9	0.0	6.3
2009, 2011	2011	Treated	9	65.0**	63.1
		Untreated	9	5.0	25.0
2009, 2010, 2011	2011	Treated	9	72.9**	68.8 **
		Untreated	9	14.6	10.4

^a^Untreated: fields where Aflasafe was never applied; Treated: fields where Aflasafe was applied (10 kg/ha, 2-weeks before flowering) during the indicated year. ^b^Frequency of Aflasafe genotypes, expressed as a percentage of the total section *Flavi* community, were log transformed (log_10_[*x* + 1]) prior to analysis to stabilize the variance. Significant differences between frequencies of Aflasafe genotypes in soil and grain from treated and untreated maize fields are indicated by *P < 0.05 or **P < 0.01 based on Student’s *t*-test.

### Recovery of individual biocontrol genotypes from soil and maize grain

In untreated maize fields, the frequency of individual biocontrol AAVs was generally low in soil (0–6.9%) and grain (0–11.8%). In contrast, treated fields had relatively a high frequency of individual biocontrol AAVs in both soil (2.5–45.8%) and grain (2.8–36.1%; Table 5). The biocontrol strain La3279 was the most recovered AAV in soil from fields either treated once, treated once and left untreated for 1 year, or left untreated for 2 years. When fields were treated during all years, Ka16127 was the most common AAV followed by La3279 (Table 5). In untreated fields, the frequency of biocontrol AAVs had no consistent trends, but La3279 was generally more frequently encountered in grain than other biocontrol AAVs when fields were treated only in the first year. Further, the frequency of individual biocontrol AAVs in soil and grain of the same regime varied. For example, Ka16127 dominated in some soils, but its frequency in the grain declined from the same fields. Also, La3279 was not detected or had a low frequency in some soils, but its frequency was high in the grain from the same fields (Table 5). When treatment was discontinued for a year, La3279 had reduced frequency in soil compared to other biocontrol AAVs. In general, when proportion of one biocontrol AAVs decreased, other biocontrol AAVs occupied those niches.

**Table 5:** Frequency of individual vegetative compatibility groups (VCGs) composing Aflasafe in soil and grain from maize fields treated with diverse Aflasafe regimens in Kaduna State, Nigeria.

Year of treatment	Year of observation	Treatment^a^	*n*	Proportion of VCG (%)^b^							
				Soil				Grain			
				Og0222	La3279	La3334	Ka16127	Og0222	La3279	La3334	Ka16127
2009	2009	Treated	9	4.9*	45.8*	4.2	16.7*	16.7*	36.1*	9.7	15.3*
		Untreated	9	0	2.1	6.9	4.2	0.7	11.1	2.8	2.8
2009	2010	Treated	9	2.5	34.4*	9.4*	17.5*	10.0*	26.2*	3.1*	3.7
		Untreated	9	0.6	3.1	1.9	1.9	0.6	3.1	0	0.6
2009	2011	Treated	9	6.9*	12.5	12.5*	8.3	2.8	12.5	15.9	4.9
		Untreated	9	0.7	6.2	2.1	5.6	4.2	11.8	7.6	4.2
2009, 2010	2010	Treated	9	5.6	27.5*	13.8	32.5*	22.5*	35.0*	4.4	8.8
		Untreated	9	0	1.3	0.6	2.5	0	0	0	0.6
2009, 2010	2011	Treated	9	12.5	0	25.0*	50.0*	12.5*	37.5*	6.3	6.3
		Untreated	9	0	0	0	0	0	0	0	6.3
2009, 2011	2011	Treated	9	15.6*	6.3	15	28.1*	3.1	35.0*	12.5	12.5
		Untreated	9	0.6	0	3.8	0.6	6.3	5.6	10	3.1
2009, 2010, 2011	2011	Treated	9	16.7*	22.9	8.3	25	4.2	22.9	27.1	14.6
		Untreated	9	2.1	4.2	2.1	6.2	4.2	0	4.2	2.1

^a^Untreated: fields where Aflasafe was never applied; Treated: fields where Aflasafe was applied (10 kg/ha, 2-weeks before flowering) during the indicated year. ^b^Frequency of individual Aflasafe genotypes, expressed as a percentage of the total *Aspergillus* section Flavi community, were log transformed (log_10_[*x* + 1]) prior to analysis to stabilize the variance. Significant differences between frequencies of individual Aflasafe genotypes in soil and grain from treated and untreated maize fields are indicated by *P < 0.05 and **P < 0.01 based on Student’s *t*-test.

## Discussion

In the US, Europe, and various SSA nations, including Nigeria, biocontrol products based on native atoxigenic *A. flavus* genotypes are approved by regulatory authorities and use of these biocontrol products allows production of commercial crops with safe aflatoxin content (Cotty et al., 2007; Dorner, 2009; Moral et al., 2020). Currently, application of these products each cropping season is the recommendation on the product label. However, farmers, researchers, donors, industry, and government officers frequently ask how often the products should be applied to provide sufficient protection. This is especially a concern in Africa where several impediments may prevent smallholder farmers from applying a product every cropping season (Ehrlich et al., 2015; Gressel and Polturak, 2018; Njoroge, 2018; Kagot et al., 2019; Pitt, 2019). In the present study, the commercial aflatoxin biocontrol product Aflasafe was applied at several intervals in multiple farmers’ fields located in the state of Kaduna, Nigeria, in an effort to generate information on how frequent biocontrol products would need to be applied to maintain sustained reductions in aflatoxin contamination. Fungal communities in soil and grain and aflatoxin content in grain from treated maize fields were compared with those of paired untreated fields. Higher proportions of biocontrol genotypes occurred in soil and grain from treated fields compared to their corresponding untreated fields, regardless of the application regimen (Figure 1). However, there were differences in carry-over of biocontrol AAVs among soil and grain of the various regimens as well as between grains from treated and untreated fields subjected to accelerated poor storage conditions comparable to farmers’ conditions. Overall, lower aflatoxin concentrations were detected in grain from treated fields both at harvest and after poor storage compared to grain from untreated fields, regardless of application frequency.

The current work is the first to contrast the frequency of atoxigenic active ingredients in maize grain from untreated fields and fields treated once per season up to 3 years under smallholder farm conditions. Season-to-season carry-over of the active ingredients in treated maize fields resulted in aflatoxin reductions in maize grain produced in subsequent years without further applications. The positive impact of carry-over lasted at least 1 year after application, but was increased by repeated treatment during one or two subsequent seasons. Until more carry-over studies are conducted under diverse field conditions, the most reliable practice for reducing aflatoxins is to apply biocontrol products every season maize is planted.

Previous studies also found that applications of atoxigenic strain-based products resulted in production of greater proportions of maize grain with aflatoxin-compliant levels (Bandyopadhyay et al., 2019; Agbetiameh et al., 2020; Senghor et al., 2020, 2021; Mahuku et al., 2022; Ola et al., 2022). In the present study, total aflatoxin content of grain produced in fields that received biocontrol treatment in either the current season or up to 2 years prior had total aflatoxin content about 9-fold lower than grain produced in fields that never received treatment. During each year of the study, aflatoxin content in untreated maize was lower than previously reported in the same area (up to 2,792 ppb; Atehnkeng et al., 2014). Evaluation of biocontrol efficacy when untreated maize fields have relatively low aflatoxin may result in reduced calculated effectiveness (i.e., only 50 to 60% in some areas in the current study). However, on a practical basis, the observed 52% reduction in some fields was sufficient to produce maize grain with acceptable aflatoxin concentrations. The greatest beneficial effect of biocontrol in the current study was revealed during the poor storage experiment. Grain from some untreated maize fields had an average of 2,634 ppb total aflatoxin compared to 90 ppb in maize grains obtained from fields treated 2 years earlier.

Biocontrol application resulted in similar fungal densities of *Aspergillus* section *Flavi* in soil and grain from treated fields as in soil or grain from untreated fields. As in other studies, treatments effectively reduced levels of aflatoxins without increases in densities of *Aspergillus* section *Flavi* in soil or grain (Bandyopadhyay et al., 2019; Agbetiameh et al., 2020; Senghor et al., 2020, 2021) and without increases in crop infection (Cotty, 1994). Fungal communities in soil and grain from biocontrol-treated fields were dominated by the *A. flavus* L-morphotype and, within the L-morphotype, biocontrol AAVs dominated the crop-associated communities. The absence of the highly toxigenic S_BG_ species in grain from fields treated for three consecutive years demonstrates the biocontrol effectiveness at excluding these highly toxigenic fungi over long periods. Regardless of treatment regimen, when the proportion of biocontrol AAVs in soil was high, the proportion of biocontrol AAVs in grain was also high (Figure 1) leading to lowered aflatoxin concentration in grain as demonstrated in this study. This aspect of biocontrol effectiveness has received little attention. Many studies measure effectiveness of application of biocontrol by examining only aflatoxin content in grains from treated and untreated fields but without quantifying the proportion of the active ingredient either in the grain or in soil and relating the proportion of active ingredients in grain to aflatoxin concentration (Weaver et al., 2015; Molo et al., 2019; Weaver and Abbas, 2019). Quantifying the proportion of the active ingredient in grain from treated and untreated fields may provide valuable information on whether the applied active ingredients are successful in colonizing soil and/or grain in certain environmental conditions (Agbetiameh et al., 2019) and replace poorly performing active ingredients from a product, if necessary.

Treatment of the maize crop only the first year but not in subsequent years generally resulted in a gradual decline in the frequency of biocontrol AAVs. Highly conducive conditions for aflatoxin formation did not occur during the present study as revealed by the ‘relatively low’ aflatoxin levels (although still unacceptable by both CODEX and EU standards of 20 ppb and 4 ppb, respectively) in grain from untreated maize fields at harvest. The term ‘relatively low’ is used above because considerably higher aflatoxin levels from the same area in different years have been reported (Atehnkeng et al., 2014). However, untreated crops in the current study were associated with a relatively high frequency of S_BG_ species, which produce large quantities of aflatoxins (Cardwell and Cotty, 2002; Probst et al., 2014). Extremely high aflatoxin levels were observed in untreated maize grain subjected to poor storage. The highly toxigenic S_BG_ species likely contributed to those high levels of contamination. Results from poor storage points to the importance of an integrated aflatoxin management system that incorporates good storage practices. In Nigeria, as well as in many countries in SSA, most maize farmers sell to intermediaries who store and market the maize. Farmers store some maize for family consumption throughout the year. The storage conditions of maize stored by different intermediaries prior to marketing and by farmers for their own consumption may be poor. Treating maize fields with biocontrol provides some protection after harvest even under this poor storage that was simulated in the current study. This observation is in agreement with previous studies reporting post-harvest benefits in treated crops (Brown et al., 1991; Atehnkeng et al., 2008b; Bandyopadhyay et al., 2019; Senghor et al., 2020, 2021). Certainly, the risk of contamination will be further reduced by efforts that improve postharvest handling (Hell et al., 2003; Waliyar et al., 2014; Seetha et al., 2017; Danso et al., 2018; Walker et al., 2018).

There is the notion that biocontrol genotypes applied during a cropping cycle may not prevail in treated fields and, therefore, will not be found in subsequent years (Molo et al., 2019). However, atoxigenic genotypes may constitute >90% of the communities 1 year after treatment and > 30% even 3 years after treatment (Cotty, 2000; Cotty et al., 2007; Weaver and Abbas, 2019). Although the current study was conducted with a multi-genotype product, the findings are in agreement with previous studies that biocontrol genotypes persist between seasons. In addition, it has been argued that recombination events between applied fungi and fungi residing in treated fields may lead to the appearance of novel genotypes (Horn et al., 2009, 2016; Olarte et al., 2012; Molo et al., 2019; Andrews et al., 2020) and that this may be a reason for not detecting the applied biocontrol genotypes (Njoroge, 2018; Pitt, 2019). Such conclusions are based on data obtained under fastidious laboratory conditions and/or microplot experiments. Those hypotheses have been tested and disproved in commercial fields only in the US with an atoxigenic biocontrol product containing a single active ingredient (Grubisha and Cotty, 2015). Painstaking work is required to appropriately monitor individual VCGs/genotypes in samples from any given area (Cotty, 1994; Atehnkeng et al., 2016; Ortega-Beltran and Cotty, 2018; Shenge et al., 2019). As mentioned above, the fate of applied atoxigenic active ingredient genotypes is not commonly monitored in either treated or untreated fields and only aflatoxin values are reported (Weaver et al., 2015; Kinyungu et al., 2019; Molo et al., 2019; Weaver and Abbas, 2019). In the current study, in the absence of follow-up treatments, biocontrol genotypes continued to be detected even one or 2 years after treatment, although frequency declined gradually over time. The observed decline in frequency should be expected as genotypes of *A. flavus* will disperse from untreated areas to treated areas and vice versa (Cotty, 2006; Bandyopadhyay et al., 2016; Weaver and Abbas, 2019). In addition to gradual migration of aflatoxin-producers into treated fields, there is the potential that adaptive differences between aflatoxin-producers and atoxigenic isolates influence long-term survival. Aflatoxin producers express high concentrations (often over 10,000 ppb) of aflatoxins in both conidia (Mehl and Cotty, 2010) and sclerotia (Garber and Cotty, 1997), asexual fruiting bodies that play important roles in dispersal and survival, and aflatoxins are potent insecticides that may deter insect grazing and reproduction (Dowd, 1988; Wicklow et al., 1994). Lack of aflatoxins in sclerotia and conidia may increase the vulnerability of atoxigenic isolates to insects and other predators and thus reduce the competitiveness of atoxigenic isolates over long periods (Cotty et al., 1994).

Aflatoxin biocontrol products containing more than one active ingredient are only registered for use in African countries (Moral et al., 2020). The registration of those 14 commercial products in Africa required reporting to regulators performance of atoxigenic individual genotypes when utilized simultaneously. However, only few of those studies have been published resulting in a dearth of revelant information accessible to the scientific community. Multi-genotype products are being pursued only in the US, where three experimental products, each containing two active ingredients have been tested in on-station trials (Molo et al., 2019) and another containing four active ingredients has been used by commercial farmers in Texas (Bhandari et al., 2020). However, the performance of individual genotypes in reducing contamination were not reported in those two studies.

Some *A. flavus* genotypes are better adapted to persistence in soil or grain (Sweany et al., 2011) while others are better able to colonize and/or sporulate on certain crops (Mehl and Cotty, 2010, 2013; Ortega-Beltran et al., 2020). This was confirmed while evaluating 12 atoxigenic AAVs native to Ghana in biocontrol formulations (Agbetiameh et al., 2019). Detection in the soil and/or grain (maize and groundnut) was used as a criterion to constitute two biocontrol products which ended up having higher efficacy than products previously reported (Agbetiameh et al., 2020). However, results of the current study suggest that reduced incidence in the grain does not necessarily mean that an atoxigenic genotype is not contributing to reduced aflatoxin content. The genotype Ka16127 was more common in soil from some treated fields than in corresponding grains. In contrast, La3279 was undetected or at low frequencies in soil from some treated fields but dominated the corresponding grains (up to 37.5%). Thus, some genotypes may be actively outcompeting aflatoxin producers in soil allowing other genotype(s) to move in higher proportions to the grain and thus have an advantage in dominating that niche. Dominance of *A. flavus* genotypes is influenced by natural founder events (Ortega-Beltran et al., 2020) but founder events are also triggered by the application of biocontrol products. It appears that depending on environmental conditions, some genotypes exploit founder events to dominate in soils. This soil dominance might provide other genotypes a numerical advantage over aflatoxin-producers while dispersing to the developing grain. Knowledge of the tendency for a genotype to dominate in soils and/or grains in certain fields, areas, or years is difficult to establish *a priori*. Use of multi-genotype products allows for some active ingredients to dominate in soil, while others compete better in grain during a given seasons’ environmental conditions. Different genotypes may vary in competitiveness in a given season at the time of treatment, during crop development, at harvest, or during the off-season, creating a balanced community of atoxigenic isolates with increased opportunity for longer-term establishment (Bandyopadhyay et al., 2022).

Efforts to further improve atoxigenic strain-based biocontrol technologies are currently underway. It is hypothesized that selecting atoxigenic genotypes with the capacity to mate with fungi residing in treated fields will create a population with low aflatoxin producing potential with higher persistence than current genotypes used in commercial products (Andrews et al., 2020). Proponents of that approach have not adequately tested the hypothesis in commercial fields, and it is not clear if this strategy would be a single application that will spread atoxigenicity through recombination or a seasonal treatment. It is also not clear how that strategy differs from current multi-genotype biocontrol strategies like the biocontrol product examined in the present study. On the other hand, the ability of fungi to mate under natural conditions is not clear (Horn et al., 2016; Luis et al., 2020). In large treated and untreated areas, *A. flavus* reproduces predominantly *via* production of conidia and recombination has been non-detectable (Grubisha and Cotty, 2010, 2015; Adhikari et al., 2016; Ortega-Beltran et al., 2016; Islam et al., 2018). Regardless, if super mating biocontrol genotypes are discovered and used, the ability of the progeny to persist in the treated field will not be dictated solely by ability to recombine.

Diverse plant pathogens disperse on both continental and global scales (Brown and Hovmøller, 2002; Isard et al., 2005; Aylor, 2018). Unquestionably, *Aspergillus* spp. disperse across several scales with the structures of communities of aflatoxin-producing fungi changing relatively rapidly across single fields, areas, and years (Bayman and Cotty, 1991; Bock et al., 2004; Ortega-Beltran et al., 2015; Ortega-Beltran and Cotty, 2018). Despite the spatiotemporal dynamism of fungal communities, in the current study a significant proportion of the applied biocontrol genotypes remained in treated fields across years but declined in frequency primarily on the local field scale. Across Nigeria, we also have found elevated levels of atoxigenic AAVs in fields treated once up to 7 years prior to sampling. The observed AAV frequencies are much higher than before introduction of the biocontrol products but still relatively low (range = 1 to 12%; A. Ortega-Beltran *unpublished*). Under both experimental and commercial field conditions, diverse biocontrol agents commonly decline in frequency a few years after their application, and even within the same year of application (Enkerli et al., 2004; Rumbos et al., 2008; Larkin, 2016; Yang et al., 2019). Climate change may also influence the long-term efficacy of biocontrol (Bandyopadhyay et al., 2016; Perrone et al., 2020), but the precise effects of climate change on the fate of biocontrol active ingredients remains to be explored.

The Harmattan season (December to March) may provide a mechanism for the declining frequency of applied atoxigenic genotypes in Nigeria and other SSA countries. The Harmattan season is a dry period in which dust blown from the Sahara Desert reaches large portions of West Africa, including virtually all of Nigeria. In 2020, the dust even reached the Americas^1^. Rather than an ephemeral, laboratory-restricted *A. flavus* sexual stage shaping the composition of *Aspergillus* communities in treated fields, a parsimonious explanation is that the applied fungi are replaced by genotypes brought from other locations during Harmattan and/or other climatic events occurring throughout the year. Atoxigenic genotypes applied during one cropping season should not be expected to remain indefinitely in treated fields, even if genotypes that are thought to recombine with the resident fungi are applied. This may in part be attributed to the adaptive differences among genotypes noted above. Even in the absence of events like the Harmattan, applied atoxigenic fungi naturally disperse beyond treatment locations (Bandyopadhyay et al., 2016). A multi-year, multi-location study revealed significant year-to-year variability in *A. flavus* community compositions in areas where biocontrol products are not used (Ortega-Beltran and Cotty, 2018). Similar rapid shifts in community composition might also be expected in areas receiving biocontrol applications. However, experience has shown that once biocontrol is used at an area-wide scale, longer-term persistence of biocontrol genotypes may occur (Cotty, 2006; Cotty et al., 2007; Jaime et al., 2017).

Although it is not possible to eliminate the risk of aflatoxin contamination in agricultural crops, it is possible to modify the population of aflatoxin-producing fungi by applying atoxigenic biocontrol genotypes. Atoxigenic genotypes used in biocontrol formulations significantly limit aflatoxin contamination both in the field and during storage (Brown et al., 1991; Doster et al., 2014; Agbetiameh et al., 2019; Senghor et al., 2020). In the current study, a single biocontrol application reduced aflatoxins over multiple years by enabling carry-over of biocontrol genotypes between years. Initial examination of the data suggests that Aflasafe should be applied for at least 2 years before treatment can be skipped for a year. However, the current study was conducted in a single agroecological zone of Nigeria. Although providing valuable information, there should be reservations in extrapolating the results to other regions. More research is needed to provide recommendations for the most cost-effective use of biocontrol products. Such recommendations should be based on farmers’ field studies where a large number of factors will determine which adjustments to the technology will have effectiveness in real-life conditions.

## Conclusion

The multi-strain biocontrol product Aflasafe is used in Nigeria to prevent aflatoxin contamination of maize and groundnut. The product label recommends one application before crop flowering every season. A frequent question is whether treatment is essential every season or if the active ingredient genotypes carry-over in soil and disperse to the next crop’s grain. The current work indicates that the active ingredients survive in soil at levels sufficient to reduce contamination of maize for up to 3 years. Carry-over of active ingredients declined over time following discontinuation of annual applications. Frequencies of active ingredients in soil correlated with presence in grain. Although greatest reductions in aflatoxin concentrations require annual applications, financial constraints may dictate that carry-over be exploited to reduce both application frequencies and associated costs. Research is needed to precisely define conditions under which application frequency may be reduced.

## Data availability statement

The raw data supporting the conclusions of this article will be made available by the authors, without undue reservation.

## Author contributions

RB and PC designed the overall projects from which data are derived. JAt, PO, JAu, PC, and RB contributed to the conception and design of the experiments. JAt, PO, JAu, and RB conducted the experiments and field studies, and collected and analyzed the data. PO, PC, and RB provided guidance. JAt and AO-B drafted the original manuscript. RB and PC secured funds for the study. All authors contributed to the article and approved the submitted version.

## Funding

The Bill & Melinda Gates Foundation (OPP1007117) and African Agriculture Technology Foundation supported this study. We also gratefully acknowledge additional funding support from the CGIAR Research Program on Agriculture for Nutrition and Health (A4NH), and the CGIAR Plant Health Initiative by CGIAR Trust Fund contributors (https://www.cgiar.org/research/).

## Conflict of interest

The authors receive no direct financial benefit from the manufacturing and marketing of the aflatoxin biocontrol product mentioned in this article. The Aflasafe name is a Trademark of the International Institute of Tropical Agriculture (IITA). IITA previously manufactured Aflasafe for use in Nigeria, Senegal, Kenya, Burkina Faso, The Gambia, and Ghana. Manufacturing and distribution responsibilities have been licensed to private or public sector entities in a few African nations. IITA charges a small licensing fee to manufacturers for use of the Aflasafe name and cost associated with technology transfer and technical backstopping. JAt, AO-B, JAu, and RB are employees of IITA.

The remaining authors declare that the research was conducted in the absence of any commercial or financial relationships that could be construed as a potential conflict of interest.

## Publisher’s note

All claims expressed in this article are solely those of the authors and do not necessarily represent those of their affiliated organizations, or those of the publisher, the editors and the reviewers. Any product that may be evaluated in this article, or claim that may be made by its manufacturer, is not guaranteed or endorsed by the publisher.

## Author disclaimer

The use of trade, firm, or corporation names in these methods is for the information and convenience of the reader. Such use does not constitute an official endorsement or approval by the USDA Agricultural Research Service, of any product or service to the exclusion of others that may be suitable. In addition, USDA-ARS makes no warranties as to the merchantability or fitness of the methodologies described on these pages for any particular purpose, or any other warranties expressed or implied. These methodologies provide a guide and do not replace published work. USDA-ARS is not liable for any damages resulting from the use or misuse of these methodologies.

## Footnotes

^1^ https://www.bbc.com/news/world-latin-america-53177693

## References

[ref1] Adhikari B. N. Bandyopadhyay R. Cotty P. J. (2016). Degeneration of aflatoxin gene clusters in *Aspergillus flavus* from Africa and North America. AMB Express 6:62. doi: 10.1186/s13568-016-0228-6, PMID: 27576895 PMC5005231

[ref2] Agbetiameh D. Ortega-Beltran A. Awuah R. T. Atehnkeng J. Cotty P. J. Bandyopadhyay R. (2018). Prevalence of aflatoxin contamination in maize and groundnut in Ghana: population structure, distribution, and toxigenicity of the causal agents. Plant Dis. 102, 764–772. doi: 10.1094/PDIS-05-17-0749-RE, PMID: 30673407 PMC7779968

[ref3] Agbetiameh D. Ortega-Beltran A. Awuah R. T. Atehnkeng J. Elzein A. Cotty P. J.. (2020). Field efficacy of two atoxigenic biocontrol products for mitigation of aflatoxin contamination in maize and groundnut in Ghana. Biol. Control 150:104351. doi: 10.1016/j.biocontrol.2020.104351, PMID: 33144821 PMC7457722

[ref4] Agbetiameh D. Ortega-Beltran A. Awuah R. T. Atehnkeng J. Islam M.-S. Callicott K. A.. (2019). Potential of atoxigenic *Aspergillus flavus* vegetative compatibility groups associated with maize and groundnut in Ghana as biocontrol agents for aflatoxin management. Front. Microbiol. 10:2069. doi: 10.3389/fmicb.2019.02069, PMID: 31555251 PMC6743268

[ref5] Amaike S. Keller N. P. (2011). Aspergillus flavus. Annu. Rev. Phytopathol. 49, 107–133. doi: 10.1146/annurev-phyto-072910-095221 21513456

[ref6] Andrews M. Carbone I. Binder A. Breakfield N. Duckworth O. Francis K.. (2020). Agriculture and the microbiome. CAST issue paper number 68. Available at: https://www.cast-science.org/wp-content/uploads/2020/08/CAST_IP68_Microbiome.pdf

[ref7] Atehnkeng J. Donner M. Ojiambo P. S. Ikotun B. Augusto J. Cotty P. J.. (2016). Environmental distribution and genetic diversity of vegetative compatibility groups determine biocontrol strategies to mitigate aflatoxin contamination of maize by *Aspergillus flavus*. Microb. Biotechnol. 9, 75–88. doi: 10.1111/1751-7915.12324, PMID: 26503309 PMC4720411

[ref8] Atehnkeng J. Ojiambo P. S. Cotty P. J. Bandyopadhyay R. (2014). Field efficacy of a mixture of atoxigenic *Aspergillus flavus* link: FR vegetative compatibility groups in preventing aflatoxin contamination in maize (*Zea mays* L.). Biol. Control 72, 62–70. doi: 10.1016/j.biocontrol.2014.02.009

[ref9] Atehnkeng J. Ojiambo P. S. Donner M. Ikotun B. Sikora R. A. Cotty P. J.. (2008a). Distribution and toxigenicity of *Aspergillus* species isolated from maize kernels from three agro-ecological zones in Nigeria. Int. J. Food Microbiol. 122, 74–84. doi: 10.1016/j.ijfoodmicro.2007.11.062, PMID: 18180068

[ref10] Atehnkeng J. Ojiambo P. S. Ikotun T. Sikora R. A. Cotty P. J. Bandyopadhyay R. (2008b). Evaluation of atoxigenic isolates of *Aspergillus flavus* as potential biocontrol agents for aflatoxin in maize. Food Addit. Contam. Part A Chem. Anal. Control. Expo. Risk Assess. 25, 1264–1271. doi: 10.1080/02652030802112635 18608502

[ref11] Ayalew A. Kimanya M. Matumba L. Bandyopadhyay R. Menkir A. Cotty P. J. (2017). “Controlling aflatoxins in maize in Africa: strategies, challenges and opportunities for improvement” in Achieving Sustainable Cultivation of Maize. Volume 2: Cultivation Techniques, Pest and Disease Control. ed. Watson D. (Cambridge, UK: Burleigh Dodds Science Publishing), 1–24.

[ref12] Aylor D. (2018). Spread of plant disease on a continental scale: role of aerial dispersal of pathogens. Ecology 84, 1989–1997. doi: 10.1890/01-0619

[ref13] Bandyopadhyay R. Atehnkeng J. Ortega-Beltran A. Akande A. Falade T. D. O. Cotty P. J. (2019). “Ground-truthing” efficacy of biological control for aflatoxin mitigation in farmers’ fields in Nigeria: from field trials to commercial usage, a 10-year study. Front. Microbiol. 10:2528. doi: 10.3389/fmicb.2019.02528, PMID: 31824438 PMC6882503

[ref14] Bandyopadhyay R. Ortega-Beltran A. Akande A. Mutegi C. Atehnkeng J. Kaptoge L.. (2016). Biological control of aflatoxins in Africa: current status and potential challenges in the face of climate change. World Mycotoxin J. 9, 771–789. doi: 10.3920/WMJ2016.2130

[ref15] Bandyopadhyay R. Ortega-Beltran A. Konlambigue M. Kaptoge L. Falade T. D. O. Cotty P. J. (2022). “Development and scale-up of bioprotectants to keep staple foods safe from aflatoxin contamination in Africa” in Microbial Bioprotectants for Plant Disease Management. eds. Köhl J. Ravensberg W. J. (Cambridge, UK: Burleigh Dodds Science Publishing), 1–41. doi: 10.19103/AS.2021.0093.16

[ref16] Bayman P. Cotty P. J. (1991). Vegetative compatibility and genetic diversity in the *Aspergillus flavus* population of a single field. Can. J. Bot. 69, 1707–1711. doi: 10.1139/b91-216

[ref17] Bhandari K. B. Longing S. D. West C. P. (2020). Soil microbial communities in corn fields treated with atoxigenic *Aspergillus flavus*. Soil Syst. 4:35. doi: 10.3390/soilsystems4020035

[ref18] Bock C. H. Mackey B. Cotty P. J. (2004). Population dynamics of *Aspergillus flavus* in the air of an intensively cultivated region of south-west Arizona. Plant Path. 53, 422–433. doi: 10.1111/j.0032-0862.2004.01015.x

[ref19] Brown R. L. Cotty P. J. Cleveland T. E. (1991). Reduction in aflatoxin content of maize by atoxigenic strains of *Aspergillus flavus*. J. Food Prot. 54, 623–626. doi: 10.4315/0362-028X-54.8.623, PMID: 31051605

[ref20] Brown J. Hovmøller M. (2002). Aerial dispersal of pathogens on the global and continental scales and its impact on plant disease. Science 297, 537–541. doi: 10.1126/science.1072678, PMID: 12142520

[ref21] Cardwell K. F. Cotty P. J. (2002). Distribution of *Aspergillus* section Flavi among field soils from the four agroecological zones of the Republic of Benin. West Africa. Plant Dis. 86, 434–439. doi: 10.1094/PDIS.2002.86.4.434, PMID: 30818721

[ref22] Chang P. K. Horn B. W. Dorner J. W. (2005). Sequence breakpoints in the aflatoxin biosynthesis gene cluster and flanking regions in nonaflatoxigenic *Aspergillus flavus* isolates. Fungal Genet. Biol. 42, 914–923. doi: 10.1016/j.fgb.2005.07.004, PMID: 16154781

[ref23] Cotty P. J. (1989). Virulence and cultural characteristics of two *Aspergillus flavus* strains pathogenic on cotton. Phytopathology 79, 808–814. doi: 10.1094/Phyto-79-808

[ref24] Cotty P. J. (1994). Influence of field application of an atoxigenic strain of *Aspergillus flavus* on the populations of *A. flavus* infecting cotton bolls and on the aflatoxin content of cottonseed. Phytopathology 84, 1270–1277. doi: 10.1094/Phyto-84-1270

[ref25] Cotty P. J. (1997). Aflatoxin-producing potential of communities of *Aspergillus* section Flavi from cotton producing areas in the United States. Mycol. Res. 101, 698–704. doi: 10.1017/S0953756296003139

[ref26] Cotty P. J. (2000). Stability of modified *Aspergillus flavus* communities: need for area-wide management. In Proceedings of the Beltwide Cotton Conference, p. 148.

[ref27] Cotty P. J. (2006). “Biocompetitive exclusion of toxigenic fungi” in The Mycotoxin Factbook: Food and Feed Topics. eds. Barug D. Bhatnagar D. Van Egdmond H. P. Van der Kamp J. W. Van Osenbruggen W. A. Visconti A. (Wageningen, the Netherlands: Wageningen Academic Publishers), 179–197.

[ref28] Cotty P. J. Antilla L. Wakelyn P. J. (2007). “Competitive exclusion of aflatoxin producers: farmer-driven research and development” in Biological Control: A Global Perspective. eds. Vincent C. Goettel N. Lazarovits G. (Wallingford: CAB International, UK), 242–253.

[ref29] Cotty P. J. Bayman P. Egel D. S. Elias K. S. (1994). “Agriculture, aflatoxins and *Aspergillus*” in The Genus Aspergillus. ed. Powell K. (New York: Plenum Press), 1–27.

[ref30] Cotty P. J. Cardwell K. F. (1999). Divergence of West African and north American communities of *Aspergillus* section Flavi. Appl. Env. Microbiol. 65, 2264–2266. doi: 10.1128/aem.65.5.2264-2266.1999, PMID: 10224034 PMC91331

[ref32] da Rocha M. Freire F. Maia F. Guedes M. Rondina D. (2014). Mycotoxins and their effects on human and animal health. Food Control 36, 159–165. doi: 10.1016/j.foodcont.2013.08.021

[ref33] Danso J. K. Osekre E. A. Opit G. P. Manu N. Armstrong P. Arthur F. H.. (2018). Post-harvest insect infestation and mycotoxin levels in maize markets in the Middle Belt of Ghana. J. Stored Prod. Res. 77, 9–15. doi: 10.1016/j.jspr.2018.02.004

[ref34] Diedhiou P. M. Bandyopadhyay R. Atehnkeng J. Ojiambo P. S. (2011). *Aspergillus* colonization and aflatoxin contamination of maize and sesame kernels in two agro-ecological zones in Senegal. J. Phytopathol. 159, 268–275. doi: 10.1111/j.1439-0434.2010.01761.x

[ref35] Donner M. Atehnkeng J. Sikora R. A. Bandyopadhyay R. Cotty P. J. (2010). Molecular characterization of atoxigenic strains for biological control of aflatoxins in Nigeria. Food Addit. Contam. Part A Chem. Anal. Control. Expo. Risk Assess. 27, 576–590. doi: 10.1080/19440040903551954, PMID: 20455156

[ref36] Dorner J. W. (2009). Development of biocontrol technology to manage aflatoxin contamination in peanuts. Peanut Sci. 36, 60–67. doi: 10.3146/AT07-002.1

[ref37] Doster M. A. Cotty P. J. Michailides T. J. (2014). Evaluation of the atoxigenic *Aspergillus flavus* strain AF36 in pistachio orchards. Plant Dis. 98, 948–956. doi: 10.1094/PDIS-10-13-1053-RE, PMID: 30708840

[ref38] Dowd P. F. (1988). Synergism of aflatoxin B1 toxicity with the co-occuring fungal metabolite kojic acid to two caterpillars. Entomol. Exp. Appl. 47, 69–71. doi: 10.1111/j.1570-7458.1988.tb02283.x

[ref39] Ehrlich K. C. Moore G. G. Mellon J. E. Bhatnagar D. (2015). Challenges facing the biological control strategy for eliminating aflatoxin contamination. World Mycotoxin J. 8, 225–233. doi: 10.3920/WMJ2014.1696

[ref40] Enkerli J. Widmer F. Keller S. (2004). Long-term field persistence of *Beauveria brongniartii* strains applied as biocontrol agents against European cockchafer larvae in Switzerland. Biol. Control 29, 115–123. doi: 10.1016/S1049-9644(03)00131-2

[ref42] Ezekiel C. N. Ortega-Beltran A. Oyedeji E. Atehnkeng J. Kössler P. Tairu F.. (2019). Aflatoxin in chili peppers in Nigeria: extent of contamination and control using atoxigenic *Aspergillus flavus* genotypes as biocontrol agents. Toxins 11:429. doi: 10.3390/toxins11070429, PMID: 31336571 PMC6669588

[ref43] Frisvad J. C. Hubka V. Ezekiel C. N. Hong S.-B. Novakova A. Chen A. J.. (2019). Taxonomy of *Aspergillus* section Flavi and their production of aflatoxins, ochratoxins and other mycotoxins. Stud. Mycol. 93, 1–63. doi: 10.1016/j.simyco.2018.06.001, PMID: 30108412 PMC6080641

[ref44] Garber R. K. Cotty P. J. (1997). Formation of sclerotia and aflatoxins in developing cotton bolls infected by the S strain of *Aspergillus flavus* and potential for biocontrol with an atoxigenic strain. Phytopathology 87, 940–945. doi: 10.1094/PHYTO.1997.87.9.940, PMID: 18945065

[ref45] Gnonlonfin G. Hell K. Adjovi Y. Fandohan P. Koudande D. Mensah G.. (2013). A review on aflatoxin contamination and its implications in the developing world: a sub-Saharan African perspective. Crit. Rev. Food Sci. Nutr. 53, 349–365. doi: 10.1080/10408398.2010.535718, PMID: 23320907

[ref46] Gressel J. Polturak G. (2018). Suppressing aflatoxin biosynthesis is not a breakthrough if not useful. Pest Manag. Sci. 74, 17–21. doi: 10.1002/ps.4694 28762637

[ref47] Grubisha L. C. Cotty P. J. (2010). Genetic isolation among sympatric vegetative compatibility groups of the aflatoxin-producing fungus *Aspergillus flavus*. Mol. Ecol. 19, 269–280. doi: 10.1111/j.1365-294X.2009.04467.x, PMID: 20025654

[ref48] Grubisha L. C. Cotty P. J. (2015). Genetic analysis of the *Aspergillus flavus* vegetative compatibility group to which a biological control agent that limits aflatoxin contamination in U.S. crops belongs. Appl. Environ. Microbiol. 81, 5889–5899. doi: 10.1128/AEM.00738-15, PMID: 26092465 PMC4551228

[ref49] Hell K. Cardwell K. F. Poehling H. M. (2003). Relationship between management practices, fungal infection and aflatoxin for stored maize in Benin. J. Phytopathol. 151, 690–698. doi: 10.1046/j.1439-0434.2003.00792.x

[ref50] Horn B. Gell R. Singh R. Sorensen R. Carbone I. (2016). Sexual reproduction in *Aspergillus flavus* sclerotia: acquisition of novel alleles from soil populations and uniparental mitochondrial inheritance. PLoS One 11, 1–22. doi: 10.1371/journal.pone.0146169, PMID: 26731416 PMC4701395

[ref51] Horn B. W. Ramirez-Prado J. H. Carbone I. (2009). Sexual reproduction and recombination in the aflatoxin-producing fungus *Aspergillus parasiticus*. Fungal Genet. Biol. 46, 169–175. doi: 10.1016/j.fgb.2008.11.004, PMID: 19038353

[ref52] Isard S. A. Gage S. H. Comtois P. Russo J. M. (2005). Principles of the atmospheric pathway for invasive species applied to soybean rust. Bioscience 55:851. doi: 10.1641/0006-3568(2005)055[0851,POTAPF]2.0.CO;2

[ref53] Islam M.-S. Callicott K. A. Mutegi C. Bandyopadhyay R. Cotty P. J. (2018). *Aspergillus flavus* resident in Kenya: high genetic diversity in an ancient population primarily shaped by clonal reproduction and mutation-driven evolution. Fungal Ecol. 35, 20–33. doi: 10.1016/j.funeco.2018.05.012, PMID: 30283498 PMC6131765

[ref54] Ismail A. Naeem I. Gong Y. Y. Routledge M. N. Akhtar S. Riaz M.. (2021). Early life exposure to dietary aflatoxins, health impact and control perspectives: a review. Trends Food Sci. Technol. 112, 212–224. doi: 10.1016/j.tifs.2021.04.002

[ref55] Jaime R. Liesner L. Antilla L. Mehl H. L. Cotty P. J. (2017). “Area-wide programs for aflatoxin mitigation: treatment to cotton can be cost effective” in National Cotton Council Beltwide Cotton Conference, January 4–6, 243–247.

[ref56] JECFA. (2018). *Safety evaluation of certain contaminants in food: Prepared by the eighty-third meeting of the Joint FAO/WHO Expert Committee on Food Additives (JECFA)*, WHO Food Additives Series, No. 74; FAO JECFA Monographs 19 bis, (Geneva: World Health Organization and Food and Agriculture Organization of the United Nations). 983

[ref57] Kagot V. Okoth S. De Boevre M. De Saeger S. (2019). Biocontrol of *Aspergillus* and *Fusarium* mycotoxins in Africa: benefits and limitations. Toxins 11:109. doi: 10.3390/toxins11020109, PMID: 30781776 PMC6409615

[ref58] Kinyungu S. Isakeit T. Ojiambo P. S. Woloshuk C. P. (2019). Spread of *Aspergillus flavus* and aflatoxin accumulation in postharvested maize treated with biocontrol products. J. Stored Prod. Res. 84:101519. doi: 10.1016/j.jspr.2019.101519

[ref59] Larkin R. P. (2016). Impacts of biocontrol products on Rhizoctonia disease of potato and soil microbial communities, and their persistence in soil. Crop Prot. 90, 96–105. doi: 10.1016/j.cropro.2016.08.012

[ref60] Leslie J. F. (1993). Fungal vegetative compatibility. Annu. Rev. Phytopathol. 31, 127–150. doi: 10.1146/annurev.py.31.090193.001015 18643765

[ref61] Luis J. M. Carbone I. Payne G. A. Bhatnagar D. Cary J. W. Moore G. G.. (2020). Characterization of morphological changes within stromata during sexual reproduction in *Aspergillus flavus*. Mycologia 112, 908–920. doi: 10.1080/00275514.2020.1800361, PMID: 32821029

[ref62] Mahuku G. Mauro A. Pallangyo B. Nsami E. Boni S. B. Koyano E.. (2022). Development of atoxigenic-based technology for biocontrol of aflatoxin in maize and groundnuts for Tanzania. World Mycotoxin J., 1–16. doi: 10.3920/WMJ2021.2758

[ref63] Mahuku G. Nzioki H. S. Mutegi C. Kanampiu F. Narrod C. Makumbi D. (2019). Pre-harvest management is a critical practice for minimizing aflatoxin contamination of maize. Food Control 96, 219–226. doi: 10.1016/j.foodcont.2018.08.032, PMID: 30713368 PMC6251936

[ref64] Matumba L. Van Poucke C. Njumbe Ediage E. De Saeger S. (2017). Keeping mycotoxins away from the food: does the existence of regulations have any impact in Africa? Crit. Rev. Food Sci. Nutr. 57, 1584–1592. doi: 10.1080/10408398.2014.993021, PMID: 25898143

[ref65] Matumba L. Van Poucke C. Njumbe Ediage E. Jacobs B. De Saeger S. (2015). Effectiveness of hand sorting, flotation/washing, dehulling and combinations thereof on the decontamination of mycotoxin-contaminated white maize. Food Addit. Contam. Part A Chem. Anal. Control. Expo. Risk Assess. 32, 960–969. doi: 10.1080/19440049.2015.1029535, PMID: 25785488

[ref66] Mehl H. L. Cotty P. J. (2010). Variation in competitive ability among isolates of *Aspergillus flavus* from different vegetative compatibility groups during maize infection. Phytopathology 100, 150–159. doi: 10.1094/PHYTO-100-2-0150, PMID: 20055649

[ref67] Mehl H. L. Cotty P. J. (2013). Nutrient environments influence competition among *Aspergillus flavus* genotypes. Appl. Environ. Microbiol. 79, 1473–1480. doi: 10.1128/AEM.02970-12, PMID: 23263958 PMC3591962

[ref68] Mehl H. L. Jaime R. Callicott K. A. Probst C. Garber N. P. Ortega-Beltran A.. (2012). *Aspergillus flavus* diversity on crops and in the environment can be exploited to reduce aflatoxin exposure and improve health. Ann. N. Y. Acad. Sci. 1273, 7–17. doi: 10.1111/j.1749-6632.2012.06800.x, PMID: 23230832

[ref69] Molo M. S. Heiniger R. W. Boerema L. Carbone I. (2019). Trial summary on the comparison of various non-aflatoxigenic strains of *Aspergillus flavus* on mycotoxin levels and yield in maize. Agron. J. 111, 942–946. doi: 10.2134/agronj2018.07.0473

[ref70] Monson M. Coulombe R. Reed K. (2015). Aflatoxicosis: lessons from toxicity and responses to aflatoxin B1 in poultry. Agriculture 5, 742–777. doi: 10.3390/agriculture5030742

[ref71] Moral J. Garcia-Lopez M. T. Camiletti B. X. Jaime R. Michailides T. J. Bandyopadhyay R.. (2020). Present status and perspective on the future use of aflatoxin biocontrol products. Agronomy 10:491. doi: 10.3390/agronomy10040491

[ref72] Narayan T. Mainville D. Geyer J. Hausdorff K. Cooley D. (2019). AgResults Impact Evaluation Report: Nigeria Aflasafe™ Challenge Project. Rockville, Maryland.

[ref73] Njoroge S. M. C. (2018). A critical review of aflatoxin contamination of peanuts in Malawi and Zambia: the past, present, and future. Plant Dis. 102, 2394–2406. doi: 10.1094/PDIS-02-18-0266-FE, PMID: 30351226

[ref74] Ojiambo P. S. Battilani P. Cary J. W. Blum B. H. Carbone I. (2018). Cultural and genetic approaches to manage aflatoxin contamination: recent insights provide opportunities for improved control. Phytopathology 108, 1024–1037. doi: 10.1094/PHYTO-04-18-0134-RVW, PMID: 29869954

[ref75] Ola O. T. Ogedengbe O. O. Raji T. M. Eze B. Chama M. Ilori O. N.. (2022). Aflatoxin biocontrol effectiveness in the real world—private sector-led efforts to manage aflatoxins in Nigeria through biocontrol-centered strategies. Front. Microbiol. 13:977789. doi: 10.3389/fmicb.2022.977789, PMID: 36118233 PMC9478371

[ref76] Olarte R. A. Horn B. W. Dorner J. W. Monacell J. T. Singh R. Stone E. A.. (2012). Effect of sexual recombination on population diversity in aflatoxin production by *Aspergillus flavus* and evidence for cryptic heterokaryosis. Mol. Ecol. 21, 1453–1476. doi: 10.1111/j.1365-294X.2011.05398.x, PMID: 22212063

[ref77] Ortega-Beltran A. Bandyopadhyay R. (2019). Comments on “trial summary on the comparison of various non-aflatoxigenic strains of *Aspergillus flavus* on mycotoxin levels and yield in maize” by M.S. Molo, et al. Agron. J.. 111: 942–946. (2019). Agron. J. doi: 10.2134/agronj2018.07.0473

[ref78] Ortega-Beltran A. Callicott K. A. Cotty P. J. (2020). Founder events influence structures of *Aspergillus flavus* populations. Environ. Microbiol. 22, 3522–3534. doi: 10.1111/1462-2920.1512, PMID: 32515100 PMC7496522

[ref79] Ortega-Beltran A. Cotty P. J. (2018). Frequent shifts in *Aspergillus flavus* populations associated with maize production in Sonora, Mexico. Phytopathology 108, 412–420. doi: 10.1094/PHYTO-08-17-0281-R, PMID: 29027887

[ref80] Ortega-Beltran A. Grubisha L. C. Callicott K. A. Cotty P. J. (2016). The vegetative compatibility group to which the US biocontrol agent *Aspergillus flavus* AF36 belongs is also endemic to Mexico. J. Appl. Microbiol. 120, 986–998. doi: 10.1016/j.funbio.2014.12.006, PMID: 26744130

[ref81] Ortega-Beltran A. Jaime R. Cotty P. J. (2015). Aflatoxin-producing fungi in maize field soils from sea level to over 2000 masl: a three year study in Sonora. Mexico. Fungal Biol. 119, 191–200. doi: 10.1016/j.funbio.2014.12.006, PMID: 25813508

[ref82] Perrone G. Ferrara M. Medina A. Pascale M. Magan N. (2020). Toxigenic fungi and mycotoxins in a climate change scenario: ecology, genomics, distribution, prediction and prevention of the risk. Microorganisms 8:1496. doi: 10.3390/microorganisms8101496, PMID: 33003323 PMC7601308

[ref83] Pitt J. I. (2019). The pros and cons of using biocontrol by competitive exclusion as a means for reducing aflatoxin in maize in Africa. World Mycotoxin J. 12, 103–112. doi: 10.3920/WMJ2018.2410

[ref84] Probst C. Bandyopadhyay R. Cotty P. J. (2014). Diversity of aflatoxin-producing fungi and their impact on food safety in sub-Saharan Africa. Int. J. Food Microbiol. 174, 113–122. doi: 10.1016/j.ijfoodmicro.2013.12.010, PMID: 24480188

[ref85] Rumbos C. Mendoza A. Sikora R. Kiewnick S. (2008). Persistence of the nematophagous fungus *Paecilomyces lilacinus* strain 251 in soil under controlled conditions. Biocontrol Sci. Tech. 18, 1041–1050. doi: 10.1080/09583150802526979

[ref86] Schreurs F. Bandyopadhyay R. Kooyman C. Ortega-Beltran A. Akande A. Konlambigue M.. (2019). “Commercial products promoting plant health in African agriculture” in Critical Issues in Plant Health: 50 Years of Research in African Agriculture. eds. Neuenschwander P. Tamò M. (Cambridge, UK: Burleigh Dodds Science Publishing), 345–363.

[ref87] Seetha A. Munthali W. Msere H. W. Swai E. Muzanila Y. Sichone E.. (2017). Occurrence of aflatoxins and its management in diverse cropping systems of Central Tanzania. Mycotoxin Res. 33, 323–331. doi: 10.1007/s12550-017-0286-x, PMID: 28785910 PMC5644708

[ref88] Senghor A. L. Ortega-Beltran A. Atehnkeng J. Callicott K. A. Cotty P. J. Bandyopadhyay R. (2020). The atoxigenic biocontrol product Aflasafe SN01 is a valuable tool to mitigate aflatoxin contamination of both maize and groundnut cultivated in Senegal. Plant Dis. 104, 510–520. doi: 10.1094/PDIS-03-19-0575-RE, PMID: 31790640

[ref89] Senghor A. L. Ortega-Beltran A. Atehnkeng J. Jarju P. Cotty P. J. Bandyopadhyay R. (2021). Aflasafe SN01 is the first biocontrol product approved for aflatoxin mitigation in two nations, Senegal and The Gambia. Plant Dis. 105, 1461–1473. doi: 10.1094/PDIS-09-20-1899-RE, PMID: 33332161

[ref90] Shenge K. C. Adhikari B. N. Akande A. Callicott K. A. Atehnkeng J. Ortega-Beltran A.. (2019). Monitoring *Aspergillus flavus* genotypes in a multi-genotype aflatoxin biocontrol product with quantitative pyrosequencing. Front. Microbiol. 10:2529. doi: 10.3389/fmicb.2019.02529, PMID: 31803149 PMC6872644

[ref91] Singh P. Callicott K. A. Orbach M. J. Cotty P. J. (2020). Molecular analysis of S-morphology aflatoxin producers from the United States reveals previously unknown diversity and two new taxa. Front. Microbiol. 11:1236. doi: 10.3389/fmicb.2020.01236, PMID: 32625180 PMC7315800

[ref92] Singh P. Cotty P. J. (2019). Characterization of Aspergilli from dried red chilies (*Capsicum* spp.): insights into the etiology of aflatoxin contamination. Int. J. Food Microbiol. 289, 145–153. doi: 10.1016/j.ijfoodmicro.2018.08.025, PMID: 30243147

[ref93] Sirma A. J. Lindahl J. F. Makita K. Senerwa D. Mtimet N. Kangethe E. K.. (2018). The impacts of aflatoxin standards on health and nutrition in sub-Saharan Africa: the case of Kenya. Glob. Food Sec. 18, 57–61. doi: 10.1016/j.gfs.2018.08.001

[ref94] Sweany R. R. Damann K. E. Jr. Kaller M. D. (2011). Comparison of soil and corn kernel *Aspergillus flavus* populations: evidence for niche specialization. Phytopathology 101, 952–959. doi: 10.1094/PHYTO-09-10-0243, PMID: 21405994

[ref95] Udomkun P. Wiredu A. N. Nagle M. Bandyopadhyay R. Müller J. Vanlauwe B. (2017a). Mycotoxins in sub-Saharan Africa: present situation, socio-economic impact, awareness, and outlook. Food Control 72, 110–122. doi: 10.1016/j.foodcont.2016.07.039

[ref96] Udomkun P. Wiredu A. N. Nagle M. Müller J. Vanlauwe B. Bandyopadhyay R. (2017b). Innovative technologies to manage aflatoxins in foods and feeds and the profitability of application – a review. Food Control 76, 127–138. doi: 10.1016/j.foodcont.2017.01.008, PMID: 28701823 PMC5484778

[ref97] USEPA. (2003). Biopesticide Registration Action Document *Aspergillus flavus* AF36. Available at: www.epa.gov/oppbppd1/biopesticides/ingredients/tech_docs?brad_006500.pdf.

[ref98] USEPA. (2016). Amendments, extensions, and/or issuances of experimental use permits - a notice by the Environmental Protection Agency on 10/05/2016. Available at: https://www.federalregister.gov/documents/2016/10/05/2016-24101/amendments-extensions-andor-issuances-of-experimental-use-permits

[ref99] USEPA. (2017). *Aspergillus flavus* AF36; amendment to an exemption from the requirement of a tolerance. Available at: https://www.gpo.gov/fdsys/pkg/FR-2017-03-22/pdf/2017-05720.pdf

[ref100] Waliyar F. Kumar P. L. Traoré A. Ntare B. R. Diarra B. Kodio O. (2014). “Pre- and postharvest management of aflatoxin contamination in peanuts” in Mycotoxins: Detection Methods, Management, Public Health and Agricultural Trade. eds. Leslie J. F. Bandyopadhyay R. Visconti A. (Oxfordshire, UK: CAB International), 209–218.

[ref101] Walker S. Jaime R. Kagot V. Probst C. (2018). Comparative effects of hermetic and traditional storage devices on maize grain: Mycotoxin development, insect infestation and grain quality. J. Stored Prod. Res. 77, 34–44. doi: 10.1016/j.jspr.2018.02.002

[ref102] Weaver M. Abbas H. K. (2019). Field displacement of aflatoxigenic *Aspergillus flavus* strains through repeated biological control applications. Front. Microbiol. 10:1788. doi: 10.3389/fmicb.2019.01788, PMID: 31447810 PMC6692475

[ref103] Weaver M. A. Abbas H. K. Falconer L. L. Allen T. W. Pringle H. L. III Sciumbato G. L. (2015). Biological control of aflatoxin is effective and economical in Mississippi field trials. Crop Prot. 69, 52–55. doi: 10.1016/j.cropro.2014.12.009

[ref104] Wicklow D. T. Dowd P. F. Gloer J. B. (1994). “Antiinsectan effects of *Aspergillus* metabolites” in *The Genus* Aspergillus*: From Taxonomy and Genetics to Industrial Applications*. eds. Powell K. A. Renwick A. Peberdy J. F. (New York: Plenum Press), 93–114.

[ref105] Wu F. (2015). Global impacts of aflatoxin in maize: trade and human health. World Mycotoxin J. 8, 137–142. doi: 10.3920/WMJ2014.1737

[ref106] Yang H. Qin C. S. Chen Y. M. Zhang G. Y. Dong L. H. Wan S. Q. (2019). Persistence of *Metarhizium* (Hypocreales: Clavicipitaceae) and *Beauveria bassiana* (Hypocreales: Clavicipitaceae) in tobacco soils and potential as biocontrol agents of *Spodoptera litura* (Lepidoptera: Noctuidae). Environ. Entomol. 48, 147–155. doi: 10.1093/ee/nvy161, PMID: 30508198

